# Mycotoxin-Containing Diet Causes Oxidative Stress in the Mouse

**DOI:** 10.1371/journal.pone.0060374

**Published:** 2013-03-28

**Authors:** Yan-Jun Hou, Yong-Yan Zhao, Bo Xiong, Xiang-Shun Cui, Nam-Hyung Kim, Yin-Xue Xu, Shao-Chen Sun

**Affiliations:** 1 College of Animal Science and Technology, Nanjing Agriculture University, Nanjing, China; 2 Laboratory of Cellular and Developmental Biology, National Institute of Diabetes and Digestive and Kidney Diseases, National Institutes of Health, Bethesda, Maryland, United States of America; 3 Department of Animal Sciences, Chungbuk National University, Cheongju, Korea; Hosptial Infantil Universitario Niño Jesús, CIBEROBN, Spain

## Abstract

Mycotoxins which mainly consist of Aflatoxin (AF), Zearalenone (ZEN) and Deoxynivalenol (DON) are commonly found in many food commodities. Although each component has been shown to cause liver toxicity and oxidative stress in several species, there is no evidence regarding the effect of naturally contained multiple mycotoxins on tissue toxicity and oxidative stress in vivo. In the present study, mycotoxins-contaminated maize (AF 597 µg/kg, ZEN 729 µg/kg, DON 3.1 mg/kg maize) was incorporated into the diet at three different doses (0, 5 and 20%) to feed the mice, and blood and tissue samples were collected to examine the oxidative stress related indexes. The results showed that the indexes of liver, kidney and spleen were all increased and the liver and kidney morphologies changed in the mycotoxin-treated mice. Also, the treatment resulted in the elevated glutathione peroxidase (GPx) activity and malondialdehyde (MDA) level in the serum and liver, indicating the presence of the oxidative stress. Moreover, the decrease of catalase (CAT) activity in the serum, liver and kidney as well as superoxide dismutase (SOD) activity in the liver and kidney tissue further confirmed the occurrence of oxidative stress. In conclusion, our data indicate that the naturally contained mycotoxins are toxic *in vivo* and able to induce the oxidant stress in the mouse.

## Introduction

Oxidative stress which refers to the *in vivo* oxidation and antioxidation imbalance reflects an imbalance between the systemic manifestation of reactive oxygen species and a biological system's ability to readily detoxify the reactive intermediates or to repair the resulting damage. In tissues, the primary antioxidant enzymes responsible for protection from oxidative stress are superoxide dismutase (SOD), catalase (CAT), and glutathione peroxidase (GPx). Superoxide anion (O_2_-) is the major free radical of aerobic respiration, which is detoxified by superoxide dismutase (SOD) catalyzing its conversion to hydrogen peroxide (H_2_O_2_), and the later is then converted to water by catalase (CAT) and glutathione peroxidase (GPx) [1].

Toxic substances which exist ubiquitously in the environment are always present in the ingredients for animal feed. Mycotoxins,one family of these toxic substances, mainly include aflatoxins (AF), zearalenone (ZEN), ochratoxins (OT), trichothecenes, and ergot alkaloids. While the toxins in the natural mycotoxins contaminated maize are composed ofAflatoxin (AF), zearalenone (ZEN), and deoxynivalenol (DON). These mycotoxins are produced from *Asper-gillus flavus, Aspergillusparasiticus, Fusarium graminearum, Fusarium culmorum,* and *Fusarium roseum* if harvesting takes place during a rainy season or if poor storage conditions are present [2]–[4], and they have tissue toxicity, reproduction toxicity and carcinogenicity. The main organs for the metabolism of mycotoxins are liver and kidney [5]. Their antioxidant activity can be changed when feeding with overdoses of mycotoxins [6].

Previous work showed that depression of growth caused by AF could remit by the antioxidants on chicken [7]. Consistently, the specific activities of hepatic enzymes of presumed importance to AF-B_1_ detoxification were significantly elevated by antioxidants on rats [8], [9],and the erythrocyte GPx and SOD activities were decreased significantly in testis of male rats fed with an AF contaminated diet [10]. For ZEN, in addition to the estrogen function, it also could cause tissue oxidative stress. The main targets of ZEN were those tissues enriched in the estrogen receptors, liver, kidney and immune systems [11], [12]. ZEN was found to have hepato nephrotoxicity and could disturb the enzymatic and hematological parameters of mice within 48 h taken orally [13]. DON is the secondary metabolites produced by *Fusarium*, similar to ZEN. Enzymatic or non-enzymatic reactions can scavenge DON-generated reactive oxygen species (ROS), subsequently preventing oxidative stress [14]. Diets contaminated with DON and ZEA at 8.3 mg/kg were able to induce the oxidative stress and compromise the blood phagocyte activity in fattening chicken [15]. Also, activity of blood SOD significantly reduced in the DON-treated group (3 mg/kg) of broilers in comparison to the control [16].

Although the existing studies on pig and chicken have shown that tissue oxidative stress could be induced by the individual ZEN and DON treatment [4], [17], [18], most of work were based on the separated mycotoxin treatment. Compared to pure mycotoxin, multiple mycotoxins contained in the naturally contaminated feed have more fierce toxicity and include the synergism and antagonism. Therefore, the aim of this study is to investigate the regulation of multiple mycotoxins on the oxidative stress using an *in vivo* model. In contrast to the pure mycotoxin feeding experiments, consumption of naturally contaminated multiple mycotoxins *in vivo* could provide more actual information on the effects of mycotoxins on the tissue toxicity and oxidative stress.

## Materials and Methods

### Ethic statement

Animal care and use were conducted in accordance with the Animal Research Institute Committee guidelines of Nanjing Agricultural University, China. Mice were housed in a temperature-controlled room with proper darkness-light cycles, fed with a regular diet, and maintained under the care of the Laboratory Animal Unit, Nanjing Agricultural University, China. The mice were killed by cervical dislocation. This study was specifically approved by the Committee of Animal Research Institute, Nanjing Agricultural University, China.

### Animals and feed treatment

The female 4-week-old ICR mice were kept at a constant temperature of 24°C ±2°C in a 12 hr light/dark cycle and had unrestricted access to food and water throughout the period of the study. Mice were randomly assigned to 3 groups (n = 15) and placed on the diets for 4 weeks. Organ weights were recorded at the week of 7. And then, all the mice were sacrificed after 12 h of fasting. Blood samples were collected for biochemical assays. The liver and the kidney were removed and weighted after rinsed with ice-cold saline.

Feed ingredients used in formulating the control diet did not contain mycotoxins at detectable levels. The natural mycotoxins contaminated maize was obtained from a Wens Breeding Groups (Suzhou, China), which was discarded completely due to severe mold growth. The contaminated diet treatments were formulated by replacing mycotoxin-free maize with natural mycotoxins contaminated maize. The mycotoxins contaminated maize contains AF 597 µg/kg, ZEN 729 µg/kg, DON 3.1 mg/kg. Low level group and high level group feeds were added with percentages of 5% and 20% natural mycotoxins contaminated maize.

### Sample collection

After obtaining the weight of the mice, blood samples were collected from orbital venous plexus. The livers and kidney were dissected out immediately and washed with normal saline, dried on a filter paper and weighted. Then the livers and kidney were prepared immediately for further examinations. Liver, kidney and spleen weight index (%) were calculated as liver (kidney, spleen) weight/body weight×100. One piece of liver and right kidney were fixed in 4% paraformaldehyde, and embedded in paraffin for morphometric analysis. Paraffin embedded tissue was cut into 4 µm thick cross-sections, and sections were stained with hematoxylin and eosin.

### Biochemical assay

Blood samples were centrifuged at 3500 rpm for 15 min. Serum was collected and used to determine biochemical parameters: glutamic-oxalacetic transaminase (GOT), glutamic-pyruvic transaminase (GPT), SOD, MDA, CAT, GPx. The suspension of liver and kidney was centrifuged at 3500 rpm for 10 min, the supernatant was collected and the activities of SOD, MDA, CAT, GPx were analyzed. All operations were done at 4°C.

The activities of SOD, CAT, GPx, and MDA were assayed using commercial reagent kits obtained from the Institute of Biological Engineering of Nanjing Jiancheng (Nanjing, China) [19], [20]. The MDA concentration was determined by the previous method [21] and expressed as nmol mg protein^−1^. Total proteins were quantified by the classical Bradford method with Coomassie Brilliant Blue G-250 [22]. Analyses of total superoxide dismutase (T-SOD) activity was based on SOD-mediated inhibition of nitrite formation from hydroxyammonium in the presence of O_2_^−^ generators (xanthine/xanthine oxidase) [23]. GPx activity was estimated by the analysis of reduced GSH in the enzymatic reaction [24]. The activity of serum GOT and GPT was assayed according to the method that usually used in clinical examination [25].

### Statistical analysis

The data were expressed as mean ± SE and analyzed by one-way ANOVA, followed by LSD's post hoc test, which was provided by SPSS16.0 statistical software. The level of significance was accepted as p<0.05.

## Results

### Mycotoxin causes the increased index of liver, kidney and spleen

**Figure 1:**
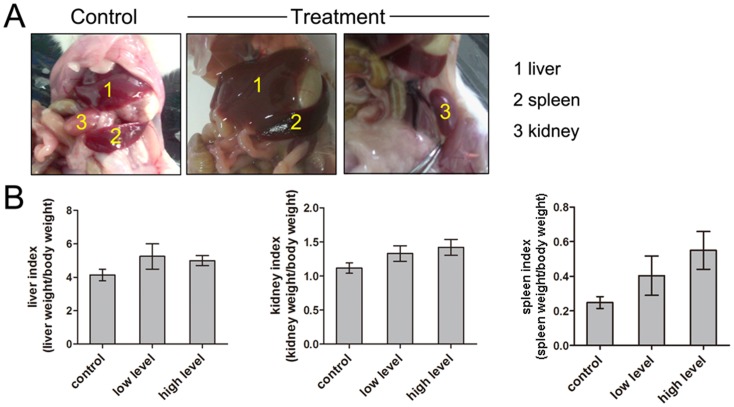
Effect of mycotoxin contaminated diet on the organ pathology changes and organ index in mouse. (A) Liver, spleen and kidney morphology changes in the treatment group observed by naked eye showing hyperemia, swelling and cirrhosis. (B) Liver index, kidney index and spleen index of treatment increased compared to the control.

After four-week diet, the model had been successfully set up, and the activities of mice in the treatment group exhibited obviously depressed compared to the control group (data not shown). As shown in the organ pathology pictures, hyperemia, swelling and cirrhosis were found in liver, kidney and spleen of treated mice (Fig. 1A). The organ index was also increased after dietary treatment. In the control group, the liver index was 4.14±0.35%, while after treatment, the liver index raised to 5.25±0.76% and 5.00±0.30% for the low level and high level groups, respectively. Similar results were observed in the kidney and spleen: Kidney index increased from 1.12±0.08% to.1.33±0.11% and 1.42±0.12%; Spleen index rose from 0.25±0.03% to 0.40±0.11% and 0.55±0.11% (Fig. 1B).

### Mycotoxin cause the liver and kidney histopathological change

**Figure 2:**
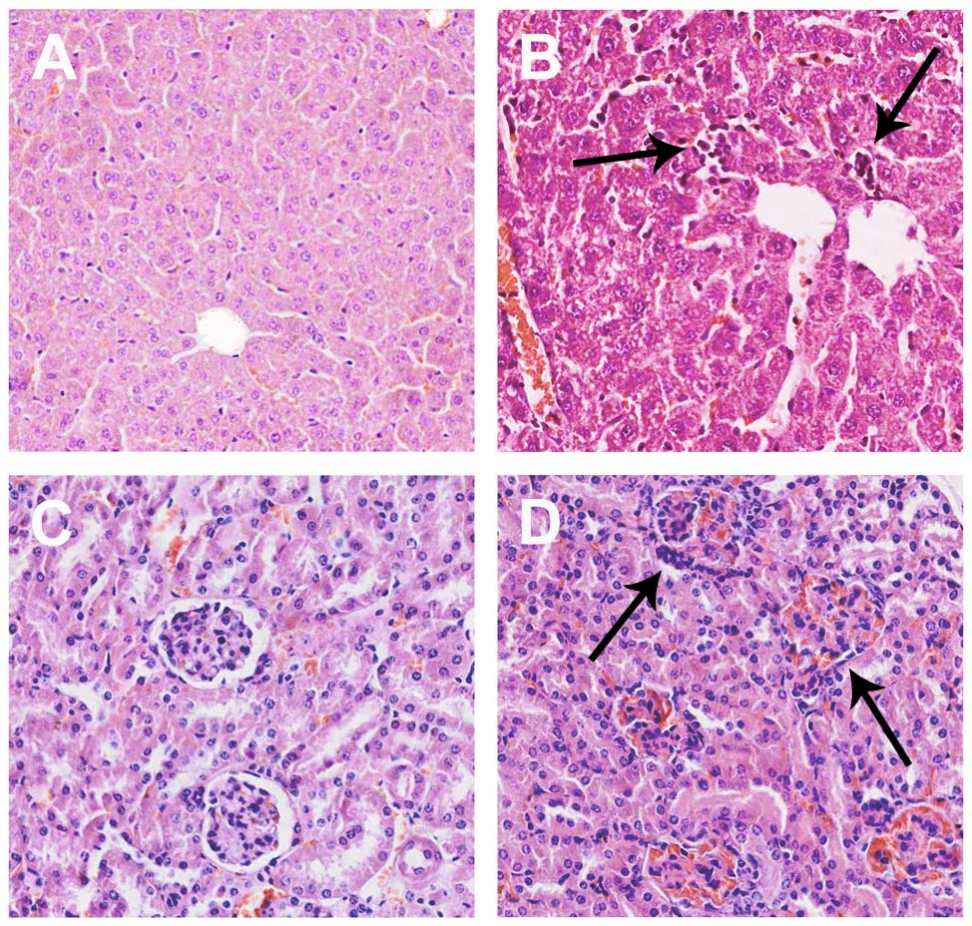
Hemotoxylin and eosin stained sections of mice livers and kidneys: (A) Normal liver section; (B) liver section of multiple mycotoxin-treated mice. The arrowheads display the apoptotic cells. (C) Normal kidney section; (D) kidney section of multiple mycotoxin-treated mice, showing with interstitial edema, tubular dilatation in the terminal part and necrosis of epithelium in the kidney glomerulus (as the arrow showed).

The liver and kidney histopathological changes were showed in Fig.2. The control group did not show any morphological changes in the two tissues (Fig. 2A, C). By contrast, the liver sections of treatment indicated multiple mycotoxin-treated mice induced inflammation. Ballooning degeneration and hepatocyte necrosis was seen in the centrilobular zone with a mild degree of associated hemorrhage (Fig.2B). The kidneys of multiple mycotoxin-treated mice showed marked histological changes in the cortex and outer medulla. The kidney sections showed tubular brush-border loss, interstitial edema, tubular dilatation, necrosis of epithelium (Fig.2D).

### Mycotoxin causes the raise of GOT and GPT levels in the serum

**Figure 3:**
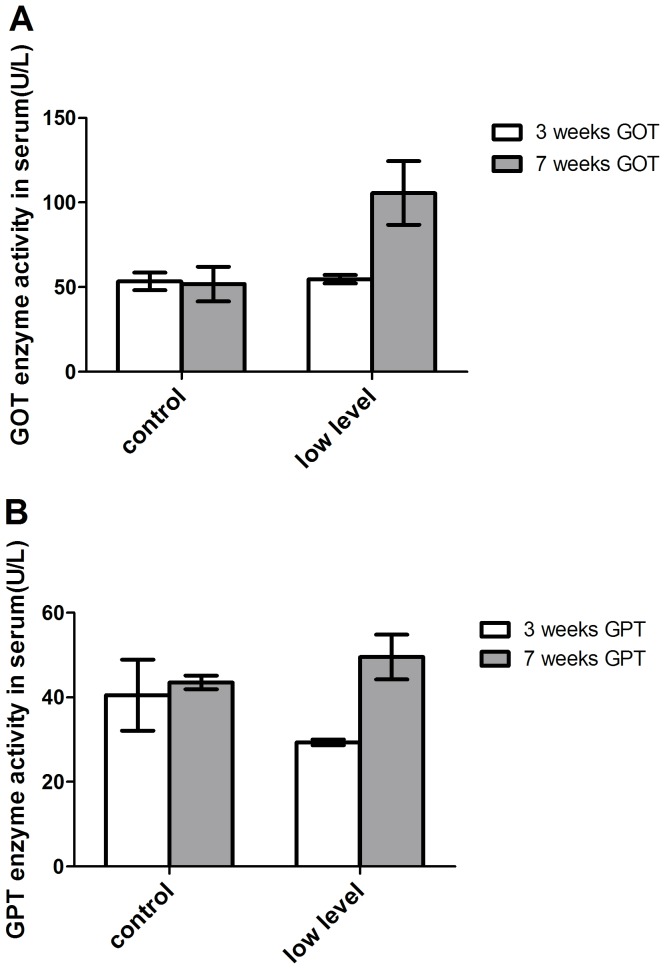
Effect of mycotoxin contaminated diet on GPT and GOT enzyme activities in the serum. (A) GPT enzyme activity in the serum was enhanced after mycotoxin consumption compared to the control group. (B) GOT enzyme activity in the serum increased after mycotoxin consumption compared to the control group.

To confirm the damage of Mycotoxin to liver, the serum GOT and GPT levels, the main parameters of liver function, were determined at week 3 and 7 of diet. The result showed that there was no difference among all groups of the GOT and GPT levels at week 3 (GOT: 53.38±5.17 U/L vs 54.68±2.45 U/L; GPT: 40.50±8.36 U/L vs 29.34±0.71 U/L). However, after consumption of mycotoxins-containing diet, the mean GOT level of the treatment group was 2-fold higher than the control group (105.6±18.79 U/L vs 51.83±10.21 U/L) (Fig. 3A). Similar result was observed in the GPT level that increased after consumption of mycotoxin contaminated diet (Control: 40.50±8.36 U/L vs 43.50±1.60 U/L; Treatment: 29.34±0.71 U/L vs 49.53±5.30 U/L) (Fig. 3B).

### Mycotoxin causes the oxidative stress in the serum

**Figure 4:**
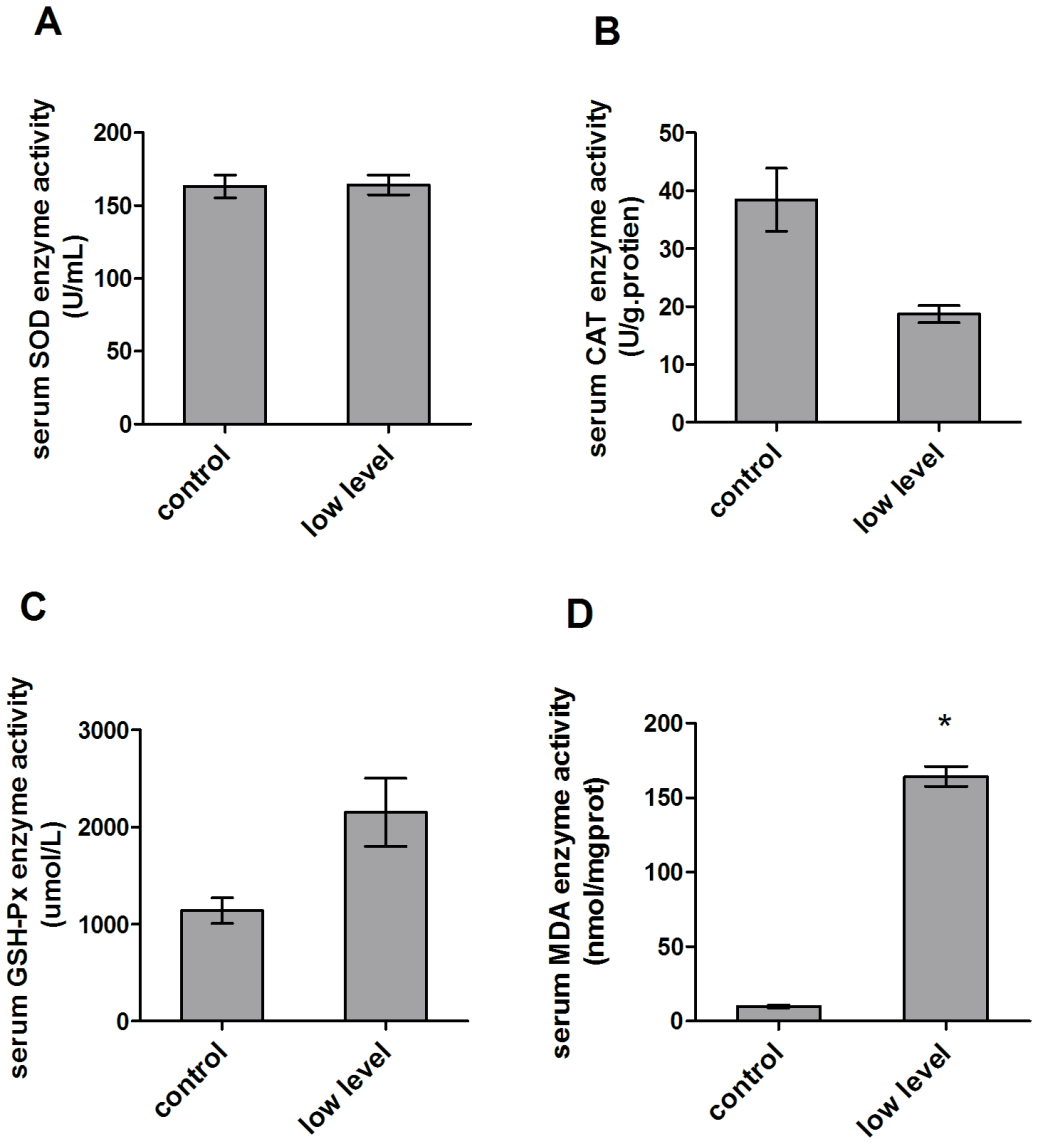
Effect of mycotoxin contaminated diet on oxidative related enzymes in the serum. (A) Mycotoxin contaminated diet did not induce the SOD activity change in the serum of treated mice. (B) Mycotoxin contaminated diet decreased the CAT activity in the serum of treated mice. (C) Mycotoxin contaminated diet enhanced the GPx activity in the serum of treated mice. (D) Mycotoxin contaminated diet caused the MDA level significantly increased in the serum of treated mice.

To determine whether the multiple mycotoxins could induce the oxidative stress *in vivo*, we first examined the SOD, CAT andGPx activities as well as the level of MDA in the serum. The results showed that SOD activity did not change (163.04±7.78 U/ml and 164.09±6.66 U/ml as the control and treatment) (Fig. 4A), but the CAT activity decreased a lot compared to the control group (38.39±5.46 U/g·protien vs 18.68±1.50 U/g·protien) (Fig. 4B). Furthermore, the multiple mycotoxins led to the increased mean GPx activity compared to the control (1140.5±131.7 umol/L vs 2149.6±349.4 umol/L) (Fig. 4C), and much higher MDA activity than the control (9.79±0.93 nmol/mgprot vs 164.09±6.66 nmol/mgprot, *p*<0.05) (Fig. 4D).

### Mycotoxin causes the oxidative stress in the liver and kidney

**Figure 5:**
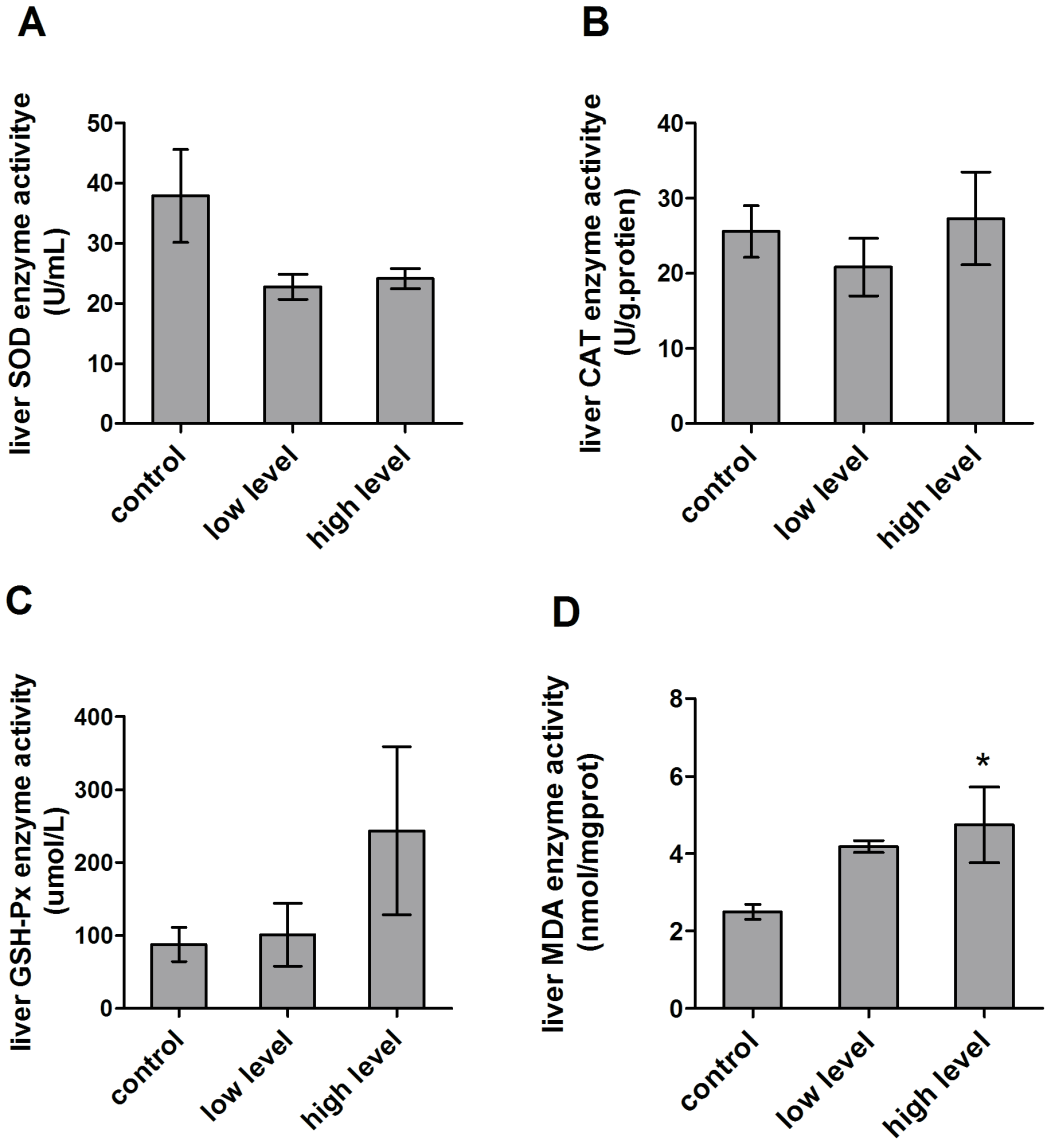
Effect of mycotoxin contaminated diet on oxidative related enzymes in the liver. (A) SOD activity was decreased after mycotoxin contaminated diet consumption compared to the control. (B) CAT activity of the treatment group decreased compared to the control group. (C) Mycotoxin contaminated diet enhanced GPx activity in the liver of treated mice. (D) Mycotoxin contaminated diet caused MDA level increased in the liver of treated mice.

Liver and kidney are the two major organs that suffer from the oxidative stress, since mycotoxins metabolism mainly occurs in the liver and the metabolites discharge in the kidney. After 4 weeks of consumption of the mycotoxin contaminated diet, SOD activity in the treatment group showed the decrease in the liver compared to the control (37.89±7.74 U/ml vs 22.76±2.07 U/ml and 24.13±1.65 U/ml) (Fig. 5A). The CAT and GPx activities showed no dose dependent (CAT:25.55±3.45 U/g·protien vs 20.84±3.83 U/g·protien and 27.30±6.18 U/g·protien; GPx: 87.65±23.37 umol/L vs 101.3±43.15 umol/L and 243.8±115.2 umol/L)(Fig. 5B, C). However, the level of MDA in the treatment group significantly increased compared to the control group (2.49±0.20 nmol/mgprot vs 4.18±0.15 nmol/mgprot and 4.74±0.98 nmol/mgprot, *p*<0.05) (Fig. 5D).

**Figure 6:**
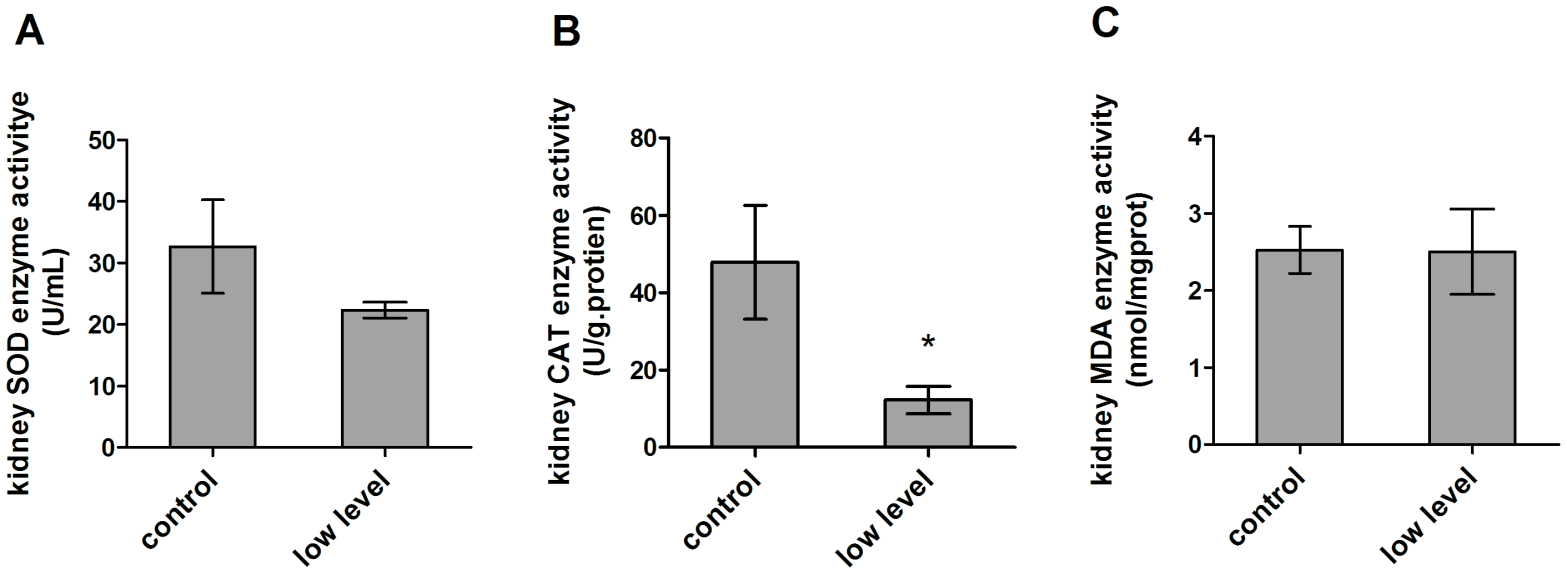
Effect of mycotoxin contaminated diet on oxidative related enzymes in the kidney. (A) Mycotoxin contaminated diet decreased SOD activity in the kidney of treated mice. (B) Mycotoxin contaminated diet caused CAT activitysignificantly decreased in the kidney of treated mice. (C) Mycotoxin contaminated diet did not induce MDA level change in the kidney of treated mice.

Next, the activity of antioxidant enzymes in the kidney was examined. As shown in Fig 5, the CAT activity in the treatment group significantly decreased from 47.85±14.73 U/g·protien to 12.25±3.56 U/g·protien compared to the control group (Fig. 6A). The mean SOD activity was also decreased from 32.67±7.60 U/ml to 22.30±1.29 U/ml (Fig. 6B). However, there was no difference for the MDA activity between the control and treatment group (2.53±0.31 nmol/mgprot and 2.50±0.55 nmol/mgprot) (Fig. 6C).

## Discussion

In the present study we examined the effects of naturally contained multiple mycotoxins on the tissue toxicity and induction of the oxidative stress in the mouse. We found that mycotoxins led to not only the hyperemia and swelling but also the increased organ index of the liver, kidney and spleen. GOT catalyzes the reversible transfer of an amino group between aspartate and glutamate, and as such, is an important enzyme in the amino acid metabolism. GOT and GPT are commonly measured clinically as a marker for the liver health, thus the enhanced GOT and GPT activities in the serum after 4 weeks consumption of mycotoxins contaminated diet indicated that multiple mycotoxins could lead to the organ pathology changes and liver damage, while the histopathological changes in liver and kidney certified this deduction.

MDA activity is an indicator of oxidative stress in both serum and liver. Previous work showed that ZEN and DON (both being 3.4 mg/kg) contaminated diets resulted in the increased level of MDA in the liver in chicken [15]. In an *in vitro* model, MDA was slightly increased when the CHO-K1 cells exposed to 50 µM ZEN [26]. Another report found that the diet containing combined DON (3 mg/kg) and Se-yeast (1 mg/kg) led to a significant reduction in MDA tissue levels [16]. Despite the controversy, our *in vivo* result showed an increase pattern with significantly elaborated MDA level in the serum and liver, which indicates that the multiple mycotoxins in the contaminated maize could lead to a worse damage than the pure mycotoxin. Moreover, the mycotoxin doses in our study were lower than what had been reported in poultry, suggesting that the rodent is more sensitive than poultry to the mycotoxins.

GPx, SOD, and CAT activities are the main parameters of anti-oxidative stress in the organism. Feeding diet contaminated with ZEN caused the decrease of antioxidant enzyme in mice [6]. Similarly, our results showed that GPx activity in the serum and liver of the mycotoxin-treated mice became higher compared to the control group, suggesting that under the oxidant stress mice are able to increase their production of GPx to get rid of the excessive H_2_O_2_ generated from the dismutation of O_2_^−^ following the administration of MDA. In the chicken, the GPx activity in mucosa and kidney was also increased when feeding a diet containing contaminated maize [17]. Additionally, it has been reported that ZEA and its metabolites lowered the levels of progesterone and oestradiol in the serum of swine, which may subsequently lead to an upregulation of selenoenzyme activities (e.g. GPx) [27]. Thus, the Glutathione-associated metabolism is a major mechanism for cellular protection against agents which generate the oxidative stress.

SOD, an important intracellular antioxidant enzyme detoxifying superoxide anion, provides a significant protection against the oxidative effect of mycotoxin contaminated maize in comparison with the GPx and CAT. The SOD activity reduced in the testicular tissue of the ZEN-treated male mice [6], [28]. While CAT has two enzymatic functions, not only catalyzing the breakdown of H_2_O_2_ into O_2_ and H_2_O, but also catalyzing the oxidation of electron donors such as ethanol or phenols in the presence of low concentrations of H_2_O_2_.In our present study, SOD activities in the liver and kidney were reduced, accompanied by the decreased CAT activity in the serum and kidney. The possible interpretation for this observation is that the metabolism of mycotoxins mainly occurs in the liver and kidney,and these three anti-oxidative enzymes cooperate as follows: SOD catalyzes the dismutation of the superoxide radical (O_2_-) to oxygen peroxide (H_2_O_2_), protecting the organs from the O_2_- damage. GPx catalyzes the reduction of H_2_O_2_ in water. In turn, CAT, another important antioxidant metalloenzyme, participates in the conversion of oxygen peroxide in water.

Taken together, the *in vivo* data presented here in mouse model demonstrate that natural multiple mycotoxins contaminated diet could result in liver damage, the elevated GPx activity in the serum and liver tissues, and the increased MDA level in the serum, indicative of oxidative stress. Moreover, the multiple mycotoxins contaminated diet may cause more serious damage than the single or pure mycotoxin.
